# The roles of iodized oil-based lymphangiography and post-lymphangiographic computed tomography for specific lymphatic intervention planning in patients with postoperative lymphatic fistula: a literature review and case series

**DOI:** 10.1186/s42155-020-00146-x

**Published:** 2020-10-21

**Authors:** F. Pan, M. Loos, T. D. Do, G. M. Richter, H. U. Kauczor, T. Hackert, C. M. Sommer

**Affiliations:** 1 Clinic for Diagnostic and Interventional Radiology, University Hospital Heidelberg, INF 110, 69120 Heidelberg, Germany; 2 Department of Radiology, Union Hospital, Tongji Medical College, Huazhong University of Science and Technology, Wuhan, China; 3 Department of General, Visceral and Transplantation Surgery, University Hospital Heidelberg, Heidelberg, Germany; 4 Clinic for Diagnostic and Interventional Radiology, Stuttgart Clinics, Katharinenhospital, Kriegsbergstrasse 60, 70174 Stuttgart, Germany

**Keywords:** Chyle, Postoperative complications, Lymphography, Computed tomography, Treatment

## Abstract

In the management of patients with postoperative lymphatic fistula (LF) in different locations, iodized oil-based lymphangiography (LAG) from trans-pedal or intranodal route is an established diagnostic approach with the potential to plan further interventional treatments. However, specific lymphatic interventions are indicated depending on different locations and morphologies of the LF. After a systematic literature review, four types of interventions can be considered, including direct leakage embolization/sclerotherapy (DLE/DLS), percutaneous afferent lymphatic vessel embolization (ALVE), percutaneous afferent lymphatic vessels disruption/sclerotherapy (ALVD/ALVS), and trans-afferent nodal embolization (TNE). In the iodized oil-based LAG, three potential lymphatic targets including confined leakage, definite afferent LVs, and definite closest afferent LNs should be comprehensively assessed. For optimal prospective treatment planning for LF, iodized oil-based post-lymphangiographic computed tomography (post-LAG CT) is a useful complement to the conventional iodized oil-based LAG, which can be performed easily after LAG. This review article summarized the current evidence of the specific lymphatic interventions in patients with postoperative LF and explored the potential benefits of post-LAG CT in the intervention planning from a case series.

## Background

Postoperative lymphatic fistula (LF) is a severe complication after thoracic, abdominal, or pelvic surgeries resulting in high mortality (Itkin et al. 2009; Baek et al. 2016a; Baek et al. 2016b; Nadolski et al. 2018). The clinical diagnosis of LF is usually based on the detection of milky fluid leakage with triglyceride > 110 mg/dl and/or positive detection of the chylomicron (Xu et al. 2011; Liu et al. 2014). In addition, iodized oil-based conventional lymphangiography (LAG) is an established diagnostic approach, in which the iodized oil such as lipiodol as a contrast medium is injected from the trans-pedal or intranodal route to visualize the lymphatic system and ruptured lymphatic vessels (LVs) (Cope and Kaiser 2002; Hur et al. 2016; Yannes et al. 2017; Reisenauer et al. 2018; Chu et al. 2019). Recently, the intranodal approach has been more recommended which is characterized by shorter examination time and easier handling compared with trans-pedal LAG (Nadolski and Itkin 2012; Nadolski et al. 2018).

**Fig. 1:**
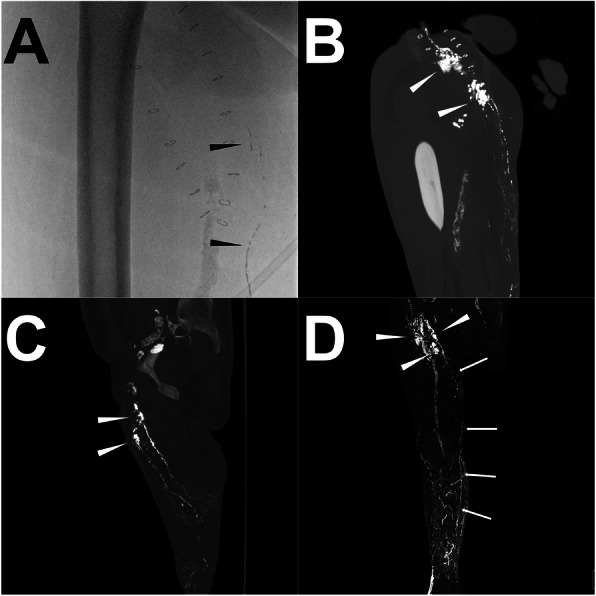
Illustration of the iodized oil-based LAG and post-LAG CT performance. **Note:** A patient with occurring persistent postoperative LF in the right groin after endovascular artery repair for an infrarenal abdominal aneurysm through the right femoral artery route. In the iodized oil-based LAG procedure, a total of 15 ml of lipiodol was injected with a velocity of 1 ml/min. However, due to the slow outflow of the lipiodol, 20 min after the accomplishment of the injection, the lipiodol only went up to the middle third of the thigh (black arrowhead) under the fluoroscopy and no obvious extravasation was found (**a**). 4 h later, the post-LAG CT was sequentially performed. The coronal (**b**) and sagittal (**c**) MIP images show definite extravasation of lipiodol (white arrowhead) at the right thigh with at least two points. The 3D-VR image (**d**) clearly visualized the major flowing route of the lymphatic fluid from calf to groin (white arrow) and the LF points (white arrowhead). Besides, abnormal lymphatic collateralization in calf could be observed (**d**). **Abbreviations:** LF – lymphatic fistula; LAG – lymphangiography; post-LAG CT – post-lymphangiographic computed tomography; MIP – maximum intensity projection; 3D-VR – three-dimensional volume rendering; LVs – lymphatic vessels

Although there are other useful diagnostic techniques such as nuclear medicine lymphoscintigraphy and gadolinium-based magnetic resonance-lymphangiography (MRL) being able to visualize the lymphatic system and LF as well, conventional iodized oil-based LAG was considered as the gold standard of reference for the prospective intervention planning for LF at different locations (Pui and Yueh 1998; Pieper et al. 2019; Pieper et al. 2020). However, the 2-dimensional feature of conventional iodized oil-based LAG can sometimes make it difficult to determine the exact anatomical information of the lymphatic system due to the overlapping structures (Ginat et al. 2009; Itkin et al. 2009; Kortes et al. 2014). Thus, post-lymphangiographic computed tomography (post-LAG CT) is recommended which can be carried out easily after iodized oil-based LAG, to illustrate better anatomical details of the lymphatic system and LF with three-dimensional spatial resolution (Ginat et al. 2009; Kawasaki et al. 2013; Kortes et al. 2014) (Fig. 1). In this review, it summarized the current evidence of the specific lymphatic interventions in patients with postoperative LF at different locations and explored the potential benefits of post-LAG CT in the intervention planning from a case series.

### Radiological demonstrations of the LF

**Fig. 2:**
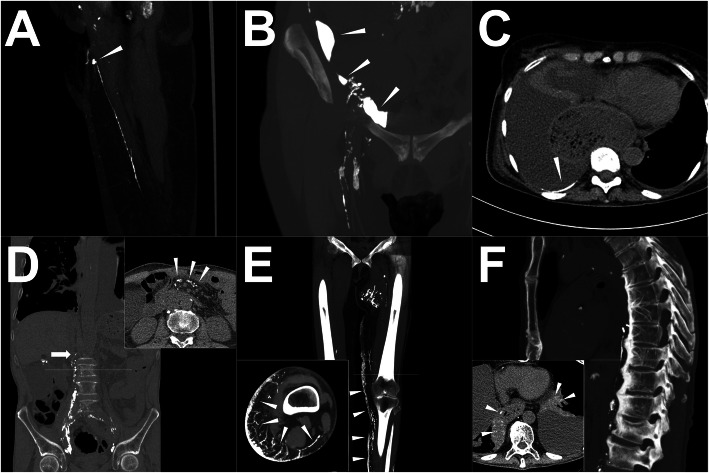
Exemplary direct and indirect demonstrations of LF. **Note: a** Local pooling demonstration of the iodized oil extravasation (white arrowhead) from the definite afferent LV; **b** Diffused distribution of the iodized oil extravasation (white arrowhead) with multiple leakage points; **c** Evidence of the iodized oil extravasation in the pleural cavity (white arrowhead) without definite LF point; **d** Abnormal distribution of iodized oil at the mesenterium (upper right corner, white arrowhead) with the disruption of main lymphatic ducts (white arrow); **e** - dermal reflux (white arrowhead) with the peripheral disruption of main lymphatic ducts; **f** - abnormal distribution of iodized oil at the lung parenchyma (bottom left corner, white arrowhead) with the iatrogenic disruption of main lymphatic ducts indicating the iodized oil from the thoracic duct directly flowed toward the lung parenchyma through abnormal lymphatic networks (so-called “PLPS”). **Abbreviations:** LF – lymphatic fistula; LV – lymphatic vessel; PLPS – pulmonary lymphatic perfusion syndrome

Above all, understanding the different radiological demonstrations of the LF is very important for prospective intervention planning. Equally, in either iodized oil-based LAG or post-LAG CT, the postoperative LF is directly characterized by the visualized iodized oil extravasation out from the afferent LVs (Fig. 2a-b), or the ectopic presence of the iodized oil in the tissue space, such as peritoneal space or pleural space without definite leaking point (Fig. 2c) (Kawasaki et al. 2013; Yoshimatsu et al. 2013). From one study, the LF with iodized oil extravasation detected by both LAG and post-LAG CT (major LF) showed a worse response to treatment than is recognized using post-LAG CT only (minor LF) (Kawasaki et al. 2013). In terms of radiological morphology, local pooling extravasation of LF (Fig. 2a) demonstrated in the post-LAG CT indicated a better prognosis than the diffused extravasation of LF (Fig. 2b) (Yoshimatsu et al. 2013). Noticeably, the typical iodized oil extravasation showed a higher density with a pronounced laser beam artifact than the normal LNs.

Except for the direct LF demonstrations, other indirect demonstrations of LF should also be noted, including iodized oil or methylene blue detected in the drainage reservoir, the disruption of main lymphatic ducts, and abnormal lymphatic collateralization and distribution (e.g. abnormal distribution of iodized oil at the lung parenchyma or mesenterium, abnormal dilated LVs, lymphatic reflux, etc.), indicating the dysfunctional LVs or potential LF after the surgery (Fig. 2d-f) (Kos et al. 2007; Savla et al. 2017). The surgery can disrupt the main lymphatic ducts, leading to central lymphatic flow disorder (CLFD) which demonstrates as the backflow of the lymph or abnormal lymphatic collateralization (Fig. 2d-e) (Savla et al. 2017). It is speculated that the occlusion of the central lymphatic duct induced a pronouncedly increased lymphatic pressure, leading to the continuous permeation of the lymph, instead of the direct rupture of the lymphatic duct. As a result, in clinical practice, patients with CLFD mostly have a worse response to the interventions (Savla et al. 2017). In addition, thoracic surgeries might result in pulmonary lymphatic perfusion syndrome (PLPS), in which the iodized oil from the thoracic duct directly flowed toward the lung parenchyma through abnormal pulmonary lymphatic networks (Savla et al. 2017) (Fig. 2f). It was probably ascribed to the development of plastic bronchitis along with the long duration of the chylothorax (Dori et al. 2016; Savla et al. 2017).

### Methods of systematic retrieve

A systematic literature review was performed in order to define eligibility criteria for further lymphatic interventions for LF after iodized oil-based LAG in accordance with the PRISMA statement (Liberati et al. 2009). The PubMed database was retrieved for previously published studies on interventional treatments for traumatic or iatrogenic lymphatic leakage based on the radiological demonstrations of iodized oil-based LAG from 05/13/1999 to 05/13/2019 (Lee et al. 2014). Due to similar anterior LAG techniques between the trans-pedal and intranodal routes, both of these two iodized oil-based LAG techniques were included in the review. The search strategy is described in the Electronic Supplement 1. Additional studies were identified through a reference check. The original articles, case series, and brief reports specifically aiming at technique description and clinical outcomes with case number ≥ 5 were included in the review. Studies were selected for inclusion by two of the authors (FP and CMS) and the decisions were made in consensus by all authors.

### A summary of specific lymphatic interventions for LF at different locations based on the prior iodized oil-based LAG

**Fig. 3:**
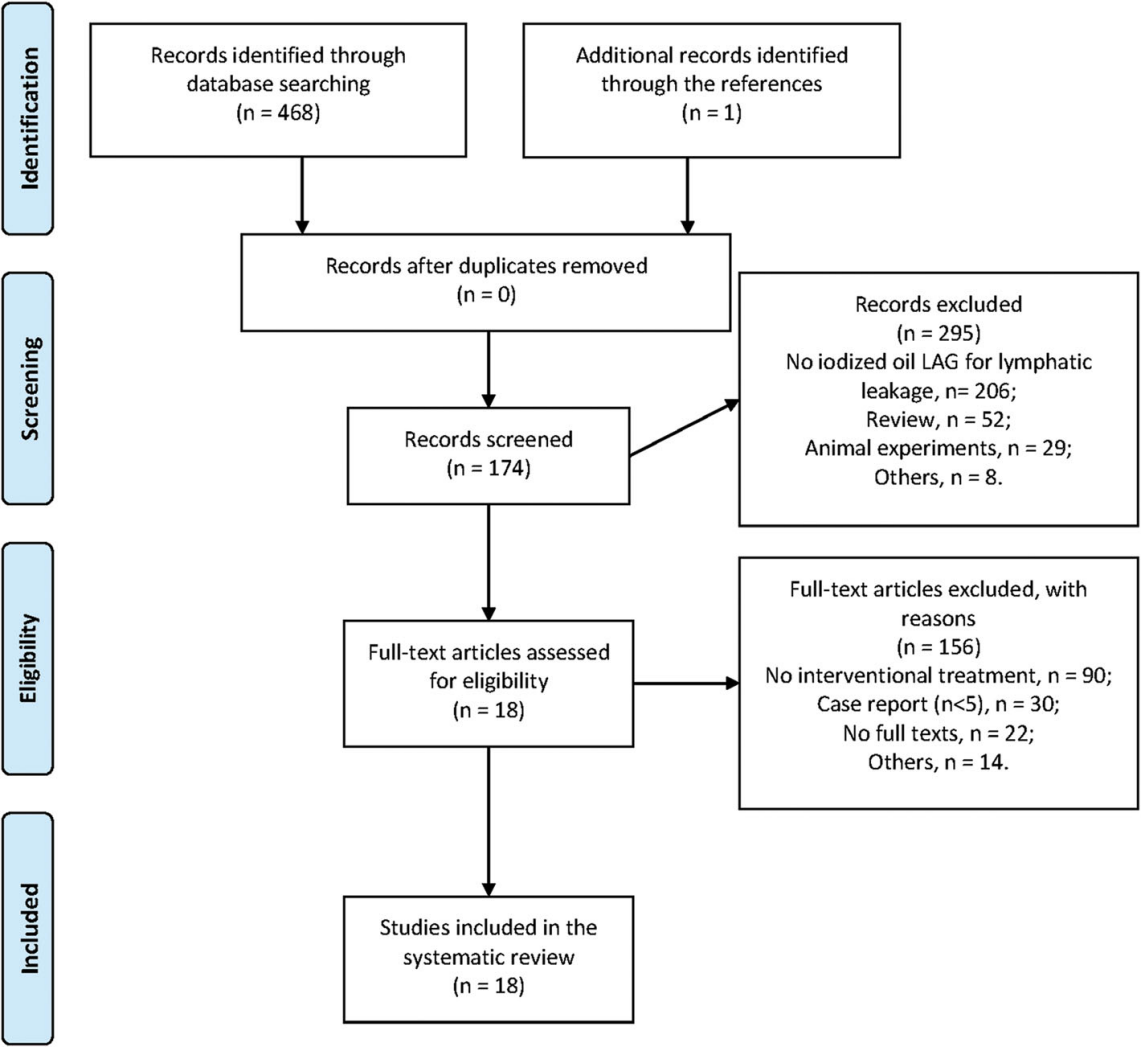
PRISMA 2009 flow diagram of the retrieved database

The details of the database retrieval are shown in Fig. 3. Finally, 18 published articles are involved (Table 1). According to the involved articles, multiple specific lymphatic interventions were developed based on the iodized oil-based LAG. Post-LAG CT was performed as a complement to LAG in only one article, which was used to identify an appropriate puncture site for further intervention (Kortes et al. 2014). Although different names were given to these interventions in the studies without unification, percutaneous embolization and disruption were the two main approaches. In summary, four major types of interventional treatments could be considered, including direct leakage embolization/sclerotherapy (DLE/DLS), percutaneous afferent lymphatic vessel embolization (ALVE), percutaneous afferent lymphatic vessels disruption/sclerotherapy (ALVD/ALVS), and trans-afferent nodal embolization (TNE) (Cope and Kaiser 2002; Boffa et al. 2008; Chen and Itkin 2011; Kortes et al. 2014; Baek et al. 2016a; Baek et al. 2016b; Hur et al. 2016; Kim et al. 2019). In most of the previously published studies, these four major types of therapeutical lymphatic interventions for LF were performed under classic fluoroscopy guidance (Table 1). However, in some studies, the CT and sonography guidance as complements to fluoroscopy guidance started to be used in the embolization and sclerotherapy, in order to reduce the ectopic injury and monitor the procedure-related complications, especially for the deep LF (Kortes et al. 2014; Hur et al. 2016; Yannes et al. 2017; Yildirim et al. 2018; Kim et al. 2019). In the following parts, these interventions were explicitly introduced:

**Table 1:** Involving published articles after systematic retrieval

Year	Author and reference	Patient number	Clinical presentation	Performance of LAG and post-LAG CT	Types of percutaneous intervention after LAG	Guidance modality	Agents	Technical success	Clinical success
2002	Cope C, et al. (Cope and Kaiser 2002)	42	Chylothorax	TL	TDE	Fluoroscopy	Coils, PVA, or NBCA.	89.6%	84.6%
					TDD		21- or 22-gauge needle		43.8%
2005	Binkert CA, et al. (Binkert, Yucel et al. 2005)	8	Chylothorax	TL	TDE	Fluoroscopy	Coils + NBCA	100.0%	100.0%
					TDD		21-gauge needle		100.0%
2008	Boffa DJ, et al. (Boffa et al. 2008)	37	Chylothorax	TL	TDE	Fluoroscopy	NBCA ± coils	57.1%	100.0%
					TDD		22-gauge needle		66.7%
2010	Itkin M, et al. (Itkin et al. 2010)	109	Chylothorax/chylopericardium/cervical chylous fistula	TL	TDE	Fluoroscopy	NBCA or Onyx ± coils	67.0%	90.1%
					TDD		21- or 22-gauge needle		72.2%
2014	Kortes N, et al. (Kortes et al. 2014)	18	Inguinal lymphocele/pelvic lymphocele/abdominal lymphatic leakage/chylothorax	TL and post-LAG CT	ALVS	CT	Ethanol	100.0%	77.8%
2016	Baek Y, et al. (Baek et al. 2016a)	5	Pelvic lymphocele	INL	ALVE	Fluoroscopy	NBCA	100.0%	75.0%
2016	Baek Y, et al. (Baek et al. 2016b)	21	Pelvic lymphatic leakage	INL	DLE	Fluoroscopy	NBCA	100.0%	85.0%
					ALVE		NBCA		
					TNE		NBCA		
2016	Hur S, et al. (Hur et al. 2016)	27	Chylothorax / chylous ascites/lymphoceles / skin fistula	INL	DLE	Fluoroscopy / Cone-beam CT	NBCA	89.3%	93.8%
					ALVE		NBCA		
					TNE		NBCA		
2017	Kariya S, et al. (Kariya, Nakatani et al. 2017)	5	Chylothorax	INL	TDE	Fluoroscopy	NBCA	100.0%	100.0%
2017	Yannes M, et al. (Yannes et al. 2017)	57	Chylothorax	INL	TDE	Fluoroscopy / CT / Sonography	NBCA + Coils	63.6%	90.5%
					TDD		21- or 22-gauge needle		41.7%
2018	Majdalany BS, et al. (Majdalany et al. 2018)	11	Chylothorax	INL	TDE	Fluoroscopy	NBCA ± Coils	37.5%	100.0%
					TDD		22-gauge needle		66.7%
2018	Nadolski GJ, et al. (Nadolski and Itkin 2018)	31	Refractory chylous ascites	TL/INL	DLE	Fluoroscopy	Coils + NBCA	100.0%	81.8%
					TNE		NBCA		
2018	Nadolski GJ, et al. (Nadolski and Itkin 2018)	50	Chylothorax	TL/INL	TDE	Fluoroscopy	Coils + NBCA, NBCA, Onyx, or autologous blood.	98.0%	91.8%
					TDD		21or 22-gauge needle		0.0%
2018	Reisenauer JS, et al. (Reisenauer et al. 2018)	97	Chylothorax	TL	TDE	Fluoroscopy	Coils, NBCA, Onyx, or Gelfoam.	47.5%	78.9%
2018	Smolock AR, et al. (Smolock et al. 2018)	10	Inguinal lymphocele and lymphorrhea	INL	TNE	Fluoroscopy	NBCA	100.0%	80.0%
2018	Yildirim IO, et al. (Yildirim et al. 2018)	13	Pelvic lymphocele	INL	DLE	Fluoroscopy / Cone-beam CT	NBCA	100.0%	88.9%
2018	Chu HH, et al. (Chu et al. 2019)	9	Pelvic Lymphocele	INL	DLE	Fluoroscopy	NBCA	100.0%	100.0%
					TNE		NBCA		
2019	Kim SW, et al. (Kim et al. 2019)	33	Pelvic lymphocele	INL	TNE	Fluoroscopy / Sonography	NBCA	100.0%	83.3%
					DLS		Ethanol		43.8%

**Abbreviation:** *ALVE* Afferent lymphatic vessel embolization, *ALVS* Afferent lymphatic vessel sclerotherapy, *DLE* Direct leakage embolization, *DLS* Direct leakage sclerotherapy, *INL* Intranodal iodized oil-based lymphangiography, *NBCA* N-butyl cyanoacrylate, *PVA* Polyvinyl alcohol, *post-LAG CT* Post-lymphangiographic computed tomography, *TDE* Percutaneous thoracic duct embolization, *TDD* Percutaneous thoracic duct disruption, *TL* Trans-pedal iodized oil-based lymphangiography, *TNE* Trans-nodal embolization

#### Direct leakage embolization/sclerotherapy (DLE/DLS)

In the DLE/DLS procedures, the lymphopseudoaneurysm or lymphocele (defined as a small or large confined lymphatic fluid collection extravasated from the ruptured LVs which was contained by the surrounding tissue) was directly targeted. The glue (e.g. a mixture of N-butyl cyanoacrylate (NBCA) and lipiodol) or the sclerosant (e.g. povidone-iodine, ethanol, talc, bleomycin, tetracycline, etc.) was directly injected to fill and/or flush this pooling extravasation in order to prevent continuous discharge from the LF (Hur et al. 2016; Chu et al. 2019). The advantage of this type of intervention could be used in any confined LF at different locations, such as thoracic, abdominal and pelvic LF, and other locations as well, as long as the LF is identified as being confined by the surrounding tissue. The potential ectopic damage to the surrounding tissue such as chemical peritonitis should be considered when performing DLS (Kortes et al. 2014; Kim et al. 2019). In such a case, DLE might be a better choice if that is possible. Compared with DLS, DLE showed about twice better clinical efficiency (88.9% versus 43.8%) (Yildirim et al. 2018, Kim et al. 2019). But for the large lymphocele, the injection of sclerosants via the percutaneous drainage tube is another option to seal the ruptured lymphatic channels when the DLE is impossible (Chu et al. 2019).

Because the puncture difficulty of the DLE/DLS procedure is relatively low, the technical success rate is almost 100%. However, the clinical success rate varied from 40% to 100%, commonly with a recurrence rate of 20–25% (Nadolski et al. 2018; Yildirim et al. 2018; Chu et al. 2019; Kim et al. 2019). This could potentially be assigned to the incapability of destroying or occluding leakage sites by using the single DLE or DLS (Shih et al. 2008; Kim et al. 2019). Thus, multiple DLE/DLS treatments can be also considered to increase the cure rate (Chu et al. 2019).

#### Percutaneous afferent lymphatic vessel embolization (ALVE)

In the ALVE procedures, the upstream afferent LV of the LF was targeted, which was mainly used in the treatment of chylothorax (Table 1). It is believed at ALVE has better efficiency than the DLE/DLS due to more reliable occlusion of the leakage site (Baek et al., 2016a, b). Two embolization techniques can be used in this method. First, the embolized agent, such as glue, is directly injected through the needle after the puncture (Baek et al., 2016a, b; Hur et al. 2016). In this technique, the coil is not recommended. Second, the micro-catheterization is performed before embolization. This technique is always used in the thoracic duct owing to the long distance between the puncture site (always cisterna chyli) to the leakage site, which is also called the thoracic duct embolization (TDE) (Cope and Kaiser 2002; Boffa et al. 2008). In TDE procedure, glue [e.g. a mixture of N-butyl cyanoacrylate (NBCA) and lipiodol] and/or coils was/were used to embolize the thoracic duct after the successful micro-catheterization approaching the leakage site. However, the puncture difficulty in ALVE is higher than the DLE/DLS with longer examination time, hence the modified ALVE technique was reported once, in that the needle penetrated both anterior and posterior walls of the afferent LV followed by injection of the glue simultaneously with the needle withdrawal (Baek et al. 2016a). This technique allowed the glue consolidated around and in the target LV resulting in the “cutting-off” inflow of lymphatic fluid to the leakage site (a so-called “cutting-off” embolization). It was suggested as a more feasible technique with an equal efficiency (Baek et al. 2016b).

From the published articles, the technical success of trans-needle embolization is probably higher than trans-microcatheter embolization. In two small-scale published studies, the technical success rate of trans-needle embolization was reported to be 100% with a clinical success rate of 75–85% (Baek et al. 2016a; Baek et al. 2016b). But in trans-microcatheter embolization, the technical success rate of 37.5–100.0% with the clinical success rate of 84.6–100.0% was reported (Cope and Kaiser 2002; Boffa et al. 2008; Itkin et al. 2009; Yannes et al. 2017; Majdalany et al. 2018). Therefore, the trans-microcatheter technique probably has better clinical efficiency and is recommended if technically feasible. However, LF with multiple afferent LVs is observed which is probably difficult to treat by using trans-microcatheter embolization, the trans-needle embolization can be chosen to embolize each afferent LV (Baek et al. 2016a). As it is similar to DLE/DLS treatment, ALVE can also be used in definite LF at different locations, such as thoracic, abdominal, and pelvic LF, etc., as long as there is the identification of accessible afferent LVs.

#### Percutaneous afferent lymphatic vessels disruption/sclerotherapy (ALVD/ALVS)

ALVD can be used to treat LF after the failure of ALVE (Cope and Kaiser 2002; Boffa et al. 2008; Itkin et al. 2010; Chen and Itkin 2011; Yannes et al. 2017; Majdalany et al. 2018; Nadolski and Itkin 2018). Furthermore, the afferent LV is the target of the intervention. According to the published studies, two disruption techniques were reported: mechanical needle disruption which was only reported in chylothorax (thoracic duct disruption, TDD) and disruption by application of sclerosant (afferent lymphatic vessel sclerotherapy, ALVS) (Cope and Kaiser 2002; Boffa et al. 2008; Itkin et al. 2010; Chen and Itkin 2011; Kortes et al. 2014; Yannes et al. 2017; Majdalany et al. 2018; Nadolski and Itkin 2018). In the TDD, the 21- or 22-gauge needles disrupted the target LVs by repeated needle punctures, probing, twisting, or to-and-fro “twiddling” motions (Cope and Kaiser 2002; Chen and Itkin 2011). Following the “blinded” destruction of the LV, the technical success rate of 100% was reported, but the clinical success rate varied from 0% to 100% (Cope and Kaiser 2002; Boffa et al. 2008; Itkin et al. 2010; Yannes et al. 2017; Majdalany et al. 2018; Nadolski and Itkin 2018). In fact, the difficulty for a complete destruction of the culprit LV remains. But in the ALVS, after the needle was advanced as close as possible to the target upstream afferent LVs, a small amount of contrast agent was injected to simulate the potential distribution following sclerosant injection under the CT guidance, which visualized the sclerotized region (Kortes et al. 2014). According to one published study, the technical success rate of ALVS was reported to be 100% with the clinical success rate of 77.8% (Kortes et al. 2014). Thus, sclerosant disruption probably has better efficiency than the mechanical needle disruption. Besides, when the needle is in position and the contrast is injected, the simulated sclerotized region can be clearly visualized under the CT guidance, which avoided ectopic destruction to important tissue or organs (Kortes et al. 2014). Thus, ALVS showed higher safety than mechanical needle disruption. Moreover, the ALVS can also be used in defined LF at different locations (Kortes et al. 2014).

#### Trans-afferent nodal embolization (TNE)

In the TNE procedures, the closest upstream afferent lymph node (LN) is punctured as the target structure. It is considered as a better alternative to ALVE in order to treat LF with multiple afferent LVs (Baek et al. 2016a). To ensure efficiency, the LN should be close to the leakage site, and thus achieving an embolization from the afferent LV to the leakage site (Hur et al. 2016; Kim et al. 2019). So far, the maximal distance between the target LN and the leakage site has not been recommended, as well as the optimal choice of the mixture ratio of NBCA and lipiodol. However, the ratio of the mixture of NBCA and lipiodol ranging from 1:2–1:9 can be flexibly chosen based on the status of the glue flow towards the leakage site (Chu et al. 2019; Kim et al. 2019). Due to the most common cause of treatment failure of multiple leakage sites, multiple afferent LNs should be embolized simultaneously, achieving larger treatment areas to prevent the leakage from the small or non-visible collateral branches in the actual clinical practice (Chu et al. 2019, Kim et al. 2019). From the published articles, the technical success rate was reported as 100% with the clinical success rate of more than 80% (Smolock et al. 2018; Chu et al. 2019; Kim et al. 2019). This method is feasible in the treatment of LF at abdominal, pelvic, inguinal, and lower extremities locations. But in the chylothorax, the distance between afferent LN and leakage of the thoracic duct is relatively long, hence TNE is not recommended. If the interventional radiologist finds a premature polymerization of the glue mixture before reaching the leak point or infeasible access of the afferent LNs, the TNE should not be continued or started, and other interventions should be considered such as the DLE/DLS (Chu et al. 2019).

### Dedicated treatment planning of specific lymphatic interventions from iodized oil-based LAG

**Fig. 4:**
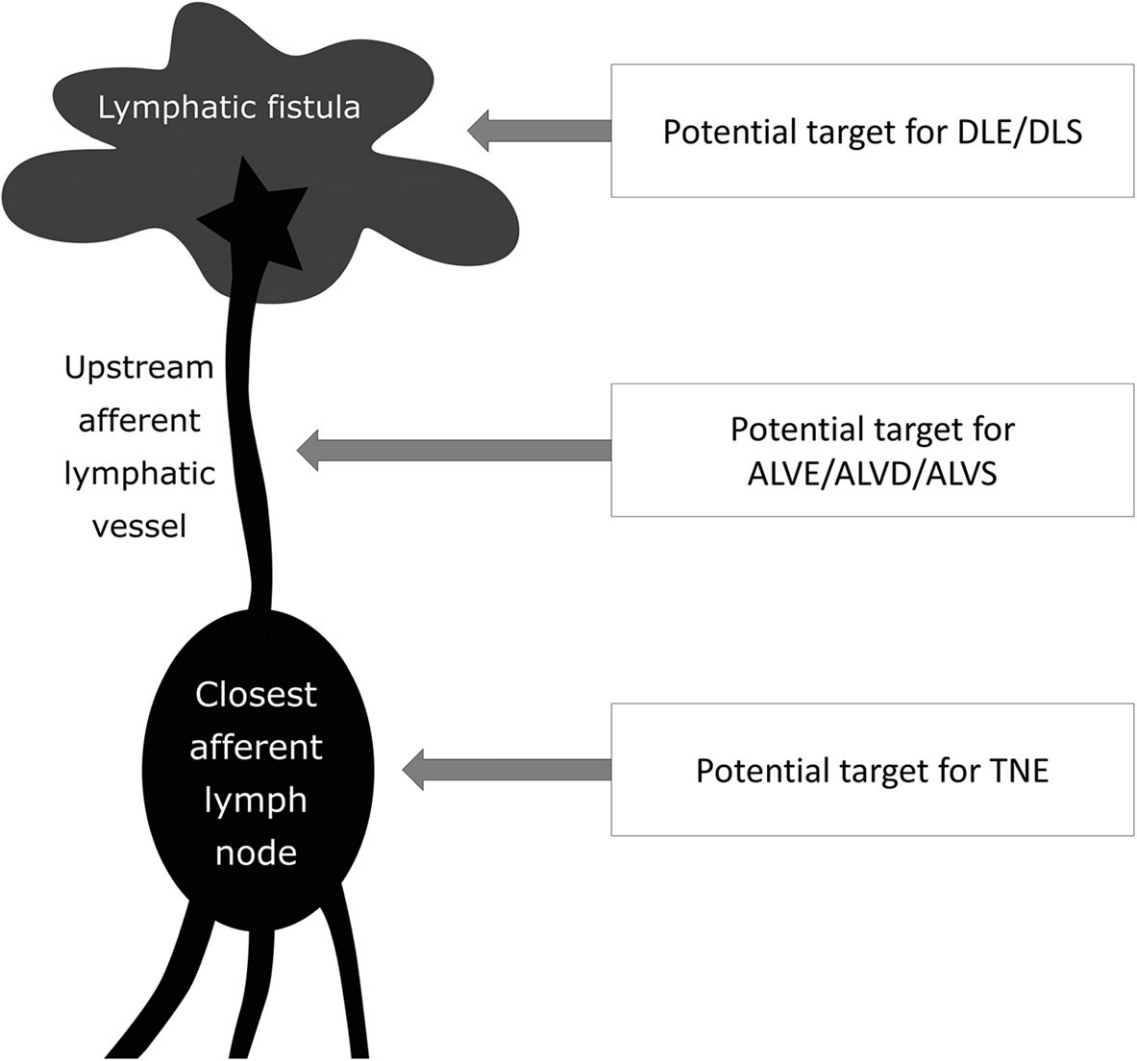
Illustration of the potential targets for different interventions

In summary, the basic indications and operations of the four major types of interventions were summarized in Table 2. As a result, three potential lymphatic targets were the major objectives for these feasible interventions, including confined leakage, definite afferent LVs, and definite closest afferent LNs (Fig. 4). In the iodized-oil based LAG, it is essential to comprehensively assess the lymphatic system and these potential targets in order to plan the optimal or feasible interventions.

**Table 2:** The four major types of therapeutical interventions based on the iodized oil-based LAG

Interventions	Basic indications	Introductions of the procedure
DLE/DLS (Hur et al. 2016, Kim et al. 2019)	Identification of confined extravasation from the LVs contained by the surrounding tissue, which could also be called “lymphopseudoaneurysm” or lymphocele.	The confined extravasation was punctured by using a 21- or 22-gauge needle, or with a drainage catheter. Then, the glue or the sclerosant solution was injected to completely fill and/or flush the confined extravasation.
ALVE (Cope and Kaiser 2002, Boffa et al. 2008, Baek et al. 2016a, Baek et al. 2016b)	Identification of the direct upstream and accessible LV that directly extravasated.	The upstream LV of the LF was directly puncture by using a 21- or 22-gauge needle. Then, the embolized agents (eg. glue, particles, coils, etc.) were injected through the needle or advanced microcatheter to completely embolize the target LV close to the leakage site.
ALVD/ALVS (Cope and Kaiser 2002, Chen and Itkin 2011, Kortes et al. 2014)	Identification of the direct upstream LV that directly extravasated, but the target upstream vessel, that was accessible for the embolization, was very small or multi-branched. In addition, the surrounding area was not close to important blood vessels or nerves.	The 21- or 22-gauge needle was advanced as close as possible to the target upstream LVs. Then, repeated probing or injection of the sclerosant solution occurred to destroy the target LVs.
TNE (Hur et al. 2016, Kim et al. 2019)	Identification of the site of the contrast extravasation and the closest upstream LN from which efferent LV extravasated a short distance away.	The closest upstream LNs of the LF were punctured by using 21- or 22-gauge needle, and then glue was injected along with the lymphatic flow to embolize the leakage at the end.

**Abbreviation:** *DLE/DLS* Direct leakage embolization/sclerotherapy, *ALVE* Afferent lymphatic vessel embolization, *ALVD/ALVS* Afferent lymphatic vessel disruption/sclerotherapy, *TNE* Trans-nodal embolization

However, the iodized-oil based LAG can’t provide enough anatomical details of the lymphatic system, especially the surrounding soft tissues. Compared with iodized oil-based LAG, it was reported that post-LAG CT could not only be used to delineate the lymphatic flow and assess the site of LF as well, but also provide the anatomical details of the surrounding tissues (Figs. 1 and 2) (Ginat et al. 2009; Kortes et al. 2014; Lee et al. 2014). Besides, post-LAG CT has a higher sensitivity to detect the LF and leakage site with a 3-dimensional visualization compared with conventional iodized oil-based LAG (64.3%–76.9% vs. 46.2%–62.5%) (Deso et al. 2012; Kawasaki et al. 2013; Yoshimatsu et al. 2013).

Although it is still lack of enough clinical data of the post-LAG CT in the intervention planning for LF, a few case reports or small-scale studies indicated that post-LAG CT had the potential to become a useful complement to LAG in the treatment planning, such as choosing an appropriate puncture route to minimize procedure-related complications and illustrate the morphology of LF (confined or not) to identify the feasibility of DLE or DLS treatment (Ginat et al. 2009; Itkin et al. 2009; Kortes et al. 2014). Thus, post-LAG CT allows a more accurate assessment of LF, with the capability for further planning of interventional treatment modalities, which previously had only referred to conventional iodized oil-based LAG.

### Technique description of post-LAG CT

The techniques of the trans-pedal and intranodal iodized oil-based LAG are already well described in several publications in the literature (Nadolski and Itkin 2012; Kortes et al. 2014; Lee et al. 2014). However, according to our knowledge, no standard technical recommendation of post-LAG CT has been published, yet. But, in either iodized oil-based LAG (trans-pedal or intranodal) or post-LAG CT, the images of both the filling phase (opacification of the target LVs and LNs until contrast runoff toward the venous angle) and nodal phase (24 h after the initial procedure) should be documented (Kos et al. 2007; Deso et al. 2012). To improve the examination efficacy and to reduce the radiation exposure, the CT could be performed just after the contrast has reached, or is above the LF level identified by fluoroscopy (Kortes et al. 2014). Empirically, the adequate timing of fluoroscopy and post-LAG CT was mandatory, such as immediately/1–3 h/4–6 h after accomplishing trans-pedal injection of iodized oil in the lower extremity and groin/pelvis and abdomen/thorax and neck (Kortes et al. 2014, Lee et al. 2014). If there was a main lymphatic vessel obliteration or surgical ligation, the inspection timing of fluoroscopy and post-LAG CT should be appropriately prolonged to ensure the inspection of the iodized oil extravasation (Fig. 1). Nevertheless, delayed post-LAG CT acquisition in the nodal phase might have diminished anatomical details of the LVs as the contrast agent had been drained out from the LVs although the iodized oil extravasation still could be detected (Dong et al. 2018).

Regarding the set-up of the CT acquisition parameters, it could be performed using the standard non-contrasted scanning protocol according to the different body parts (e.g. 120 kV_p_ with adapted tube current for thorax). The reconstruction of slice thickness and increment was mostly set up as 1 or 2 mm for better spatial resolution (Ginat et al. 2009; Safar et al. 2011; Yamada et al. 2017; Zhang et al. 2017). Except for the axial images, maximum intensity projection (MIP) reformatted imaging with three axes orientations and 3-dimensional volume rendering (3D-VR) images could facilitate to visualize the whole lymphatic vessels and LF (Fig. 1) (Ginat et al. 2009; Yamada et al. 2017). As an alternative to a conventional CT scan, a simultaneous cone-beam CT scan can also be used, which could be performed with fluoroscopy simultaneously (Yildirim et al. 2018).

### Examples of the utility of post-LAG CT in planning intervention and treatment

#### Case 1 (DLS procedure)

**Fig. 5:**
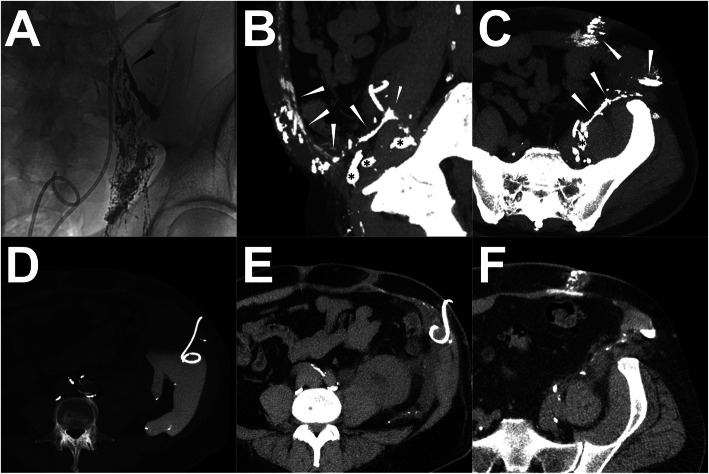
A patient underwent a DLS procedure after post-LAG CT. **Note:** Conventional iodized oil-based LAG showed the extravasation (black arrowhead) from the left iliac LVs (**a**). From the sagittal (**b**) and axial MIP (**c**) images of post-LAG CT is demonstrated an obvious LF (white arrowhead) from the iliac LVs into the peripheral lymphocele around the transplanted kidney. The afferent LNs could be identified (*). A pigtail drainage catheter was inserted into the lymphocele before the local sclerotherapy (**d**). After running out of all the lymphatic fluid, 15 ml of 95% Ethanol as the sclerosant was injected to flush the lymphocele and then this was drawn out twice. Eight (8) days after DLS, the CT scan revealed very limited residual lymphatic fluid (**e, f**). **Abbreviations:** DLS – direct leakage sclerotherapy; post-LAG CT – post-lymphangiographic computed tomography; LAG – lymphangiography; LVs – lymphatic vessels; MIP – maximum intensity projection; LF – lymphatic fistula; LNs – lymph nodes

A patient was diagnosed with a terminal renal insufficient injury caused by IgA nephropathy (Berger’s disease). After the allograft renal transplantation, a progressive lymphocele surrounding the transplanted kidney occurred. The conventional trans-pedal LAG was performed and 9 ml lipiodol was injected, with a subsequent post-LAG CT acquisition after 5 h. CT images showed clear extravasation of lipiodol from the iliac lymphatic ducts into the lymphocele. However, multiple and filiform afferent vessels were detected and hence, the ALVS and ALVE were not possible. In addition, three afferent LNs could be considered as potential targets, but the closest LN was located dorsally of the kidney which is an obstacle for puncture. Thus, the TNE was not optimal either. Eight days later, due to a lack of improvement of the lymphocele, DLS was performed. In the procedure, a pigtail drainage catheter was inserted into the lymphocele. After the drainage of lymphatic fluid, 15 ml of 95% Ethanol as a sclerosant was injected to flush the lymphocele twice. The drainage amount was monitored and ceased after 1 week. Eight days after the procedure, the CT scan revealed very limited residual lymphatic fluid, hence the drainage catheter was removed. The details are shown in Fig. 5.

#### Case 2 (ALVE procedure)

**Fig. 6:**
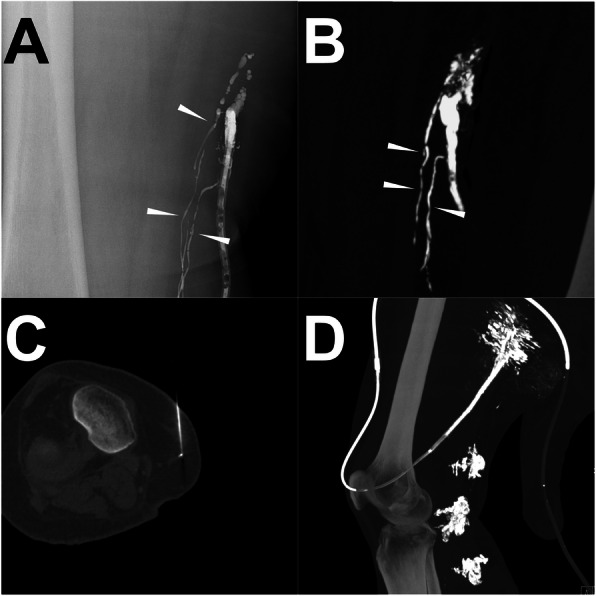
A patient underwent ALVE procedure after post-LAG CT. **Note:** Conventional iodized oil-based LAG showed the definite extravasation from three afferent LVs into the drainage (**a**). A similar demonstration was observed from the sagittal MIP images of post-LAG CT (**b**). The clear afferent LVs are shown (white arrowheads). The afferent LVs were punctured using the 21-G needles under the CT guidance penetrating the anterior and posterior walls (**c**). After the “cutting-off” embolization by using the histoacryl/lipiodol mixture (2:3) (**d**). The LF ceased immediately. **Abbreviations:** ALVE – afferent lymphatic vessel embolization; post-LAG CT – post-lymphangiographic computed tomography; LAG – lymphangiography; LVs – lymphatic vessels; LF – lymphatic fistula

In a patient after femoral lifting surgery, persistent LF at the right thigh occurred with the lymph output of 400 ml per day. Thus, the iodized oil-based LAG was performed and 16 ml of lipiodol was injected. One hour later, a sequential post-LAG CT scan was performed. The conspicuous extravasation of the lipiodol from multiple LVs draining into the drainage catheter was observed in both of the iodized oil-based LAG and post-LAG CT. One day later, the ALVE was performed. After localization of the target afferent LVs from the visualization of the residual lipiodol with the assistance of the previous post-LAG CT images, the optimal figure was achieved with four-needle puncture under CT guidance. Afterwards, a total of 11 ml histoacryl/lipiodol mixture (2:3) was injected into the LVs using the “cutting-off” technique mentioned above. After the ALVE, the LF ceased immediately and no recurrence was found in the follow-up. The details are shown in Fig. 6.

#### Case 3 (ALVS procedure)

**Fig. 7:**
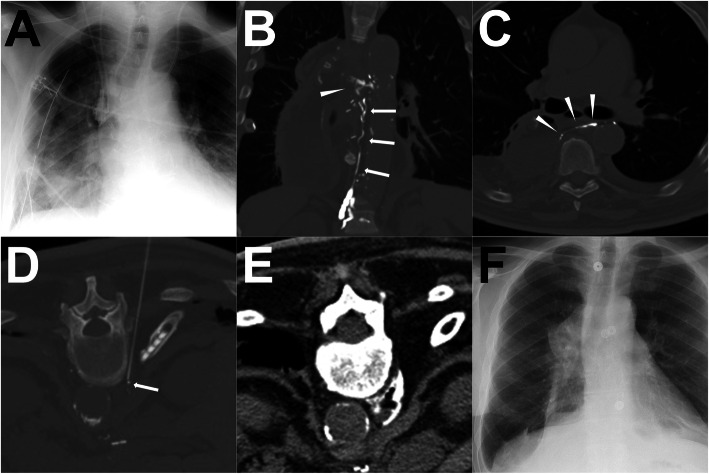
A patient underwent ALVS procedure after post-LAG CT. **Note:** After the surgery, the right chylothorax appeared with hyperdensity of the right lower lung in the chest radiogram (**a**). The coronal MIP images of post-LAG CT demonstrated a definite rupture (white arrowhead) of the thoracic duct (white arrows) and the extravasation to the right pleural cavity (**b**). The axial MIP image demonstrated a clear rupture of the thoracic duct (**c**). The lower thoracic duct was regarded as the target afferent LV for the ALVS and a 21-G Chiba needle was used to puncture close to the lower thoracic duct (**d**). Afterwards, 1 ml contrast followed with 4.0 ml 95% Ethanol being injected. The post-ALVS CT scan showed an ideal distribution of the sclerosant around the target thoracic duct (**e**). Eleven (11) days after ALVS, the chest radiogram shows no obvious recurrence of the chylothorax (**f**). **Abbreviations:** ALVS – afferent lymphatic vessel sclerotherapy; post-LAG CT – post-lymphangiographic computed tomography; MIP – maximum intensity projection; LV – lymphatic vessel

A patient was diagnosed with erosive esophagitis-induced esophageal stenosis confirmed by endoscopic biopsy, hence thoracic-abdominal esophagus resection with gastric tube-esophagus anastomosis was performed. After the surgery, the right chylothorax appeared with the average daily output of 1130 ml from the two thoracic drainage catheters. Due to the non-effective conservative treatment, conventional trans-pedal LAG was performed and 15.0 ml iodized oil was injected. Eight (8) hours later, the post-LAG CT scan was performed, showing a definite rupture of the thoracic duct with the extravasation to the right pleural cavity. From the post-LAG CT images, the distal cisterna chyli could be regarded as the target afferent LV for the ALVE treatment, or the distal thoracic duct could be regarded as the target afferent LV for the ALVS treatment. Eventually, the ALVS was chosen to be performed, since it is an easier technique. After the sclerotherapy by using 95% Ethanol, the pleural effusion drainage gradually reduced and finally ceased after 8 days. No recurrence was observed in the follow-up. The details are shown in Fig. 7.

#### Case 4 (TNE procedure)

**Fig. 8:**
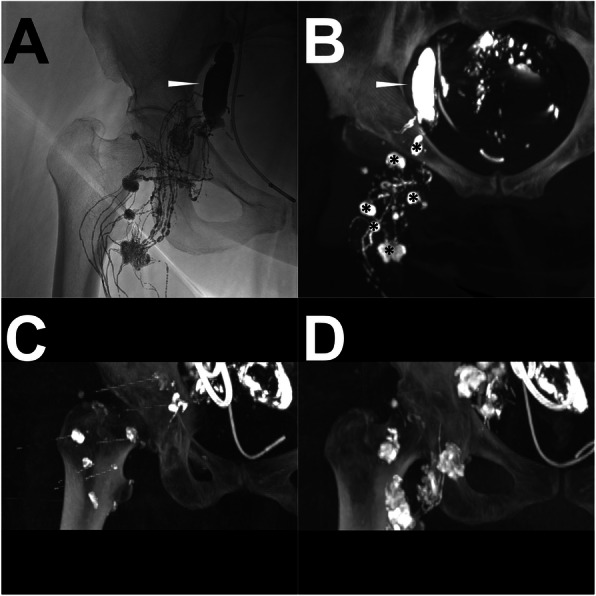
A patient underwent a TNE procedure after post-LAG CT. **Note:** The conventional iodized oil-based LAG shows the obvious extravasation (white arrowhead) from multiple afferent LVs and LNs in the right pelvis (**a**). One (1) hour later, the post-LAG CT (**b**) was sequentially performed which shows similar findings (white arrowhead) and identifies seven definite afferent LNs (*). The TNE was carried out with a CT-controlled percutaneous puncture of seven afferent LNs (**c**). After the embolization with the histoacryl/lipiodol mixture (1:1), the optimal distribution of the histoacryl/lipiodol mixture in afferent LNs and LVs is observed (**d**). **Abbreviations:** TNE – trans-afferent nodal embolization; post-LAG CT – post-lymphangiographic computed tomography; LAG – lymphangiography; MIP – maximum intensity projection; LVs – lymphatic vessels; LNs – lymph nodes

A patient was diagnosed with endometrial carcinoma after undergoing laparoscopically-assisted vaginal hysterectomy with adnexectomy. After the surgery, the LF appeared with the demonstration of extensive lymphatic fluid in the pelvis. Thus, the conventional trans-pedal LAG and sequential post-LAG CT were performed. A total of 16 ml of lipiodol was applied. Diffused extravasation with multiple afferent LVs and LNs were shown. From the three axial CT images, the multi-afferent LNs were clearly defined. Owing to the multiple and filiform afferent LVs and diffused LF, the TNE was the only choice. One month after the iodized oil-based LAG, the LF persisted. Hence, the TNE was eventually carried out. Under the CT guidance with the visualization of the target afferent LNs by the residual lipiodol, seven afferent LNs were successfully punctured. After verification of the correct needle position, sequential embolization with histoacryl/lipiodol mixture (1:1) was carried out with a total volume of 11 ml. After the TNE, the LF ceased and the pelvic lymphatic fluid disappeared after 1 week. In the follow-up, no recurrent LF was observed again. The details are shown in Fig. 8.

#### Case 5 (no intervention treatment)

**Fig. 9:**
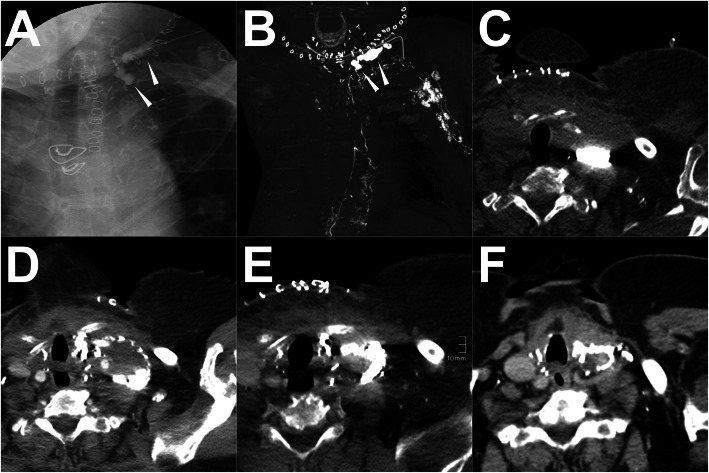
A patient underwent no interventions after iodized oil-based LAG. **Note:** Both the conventional iodized oil-based LAG (**a**) and post-LAG CT (**b**) reveal the definite rupture of the thoracic duct (white arrowhead) in the neck. In addition, the reflux of lipiodol to the left axillary and upper arm was observed which indicates the disruption of the end of the thoracic duct (**b**). In the axial post-LAG CT image, the fluid accumulation with the iodized oil extravasation is noticed (**c**). No other intervention was carried out in this patient except for the conventional iodized oil-based LAG treatment. However, with time (6d, 11d) (**d-e**), the fluid accumulation gradually reduced and the drainage eventually ceased on the 11th day. Two (2) months after LAG, the recheck of the CT scan shows no recurrence of the LF (**f**). **Abbreviations:** LAG – lymphangiography; post-LAG CT – post-lymphangiographic computed tomography

A patient was diagnosed with thyroid carcinoma while undergoing thyroidectomy. After the surgery, the persistent LF from the wound and drainage catheter occurred. Thus, the conventional trans-pedal LAG was performed with the injection of the 19 ml lipiodol, followed by the sequential post-LAG CT scan 2 h later. Both the iodized oil-based LAG and post-LAG CT images showed the typical rupture of the thoracic duct near the jugular angle with the accumulation of the lymphatic fluid in the left neck. In addition, the post-LAG CT images implied the disruption of the thoracic duct with the reflux of the lipiodol to the left upper arm. Since in this patient the main cisterna chyli and thoracic duct were very small and tortuous with multiple collaterals, it is difficult to perform either the AVLE or the TDE. Further, with the ALVS it is impossible to destroy all the afferent LVs. Thus, no further intervention was carried out. However, with the therapeutical effect of the iodized oil-based LAG, the LF gradually reduced after the procedure and ceased on the 11th day. No recurrence was found in the follow-up. The details are shown in Fig. 9.

In this case, LAG demonstrated a good therapeutical effect for LF, except for diagnostic usage. From the previous studies, iodized oil-based LAG could cure the LF with the efficacy rate of 50–70%, because iodized oil is also a classic embolized agent being capable of occluding the ruptured LVs (Matsumoto et al. 2009; Alejandre-Lafont et al. 2011; Kawasaki et al. 2013; Kortes et al. 2014; Hur et al. 2016; Yannes et al. 2017; Nadolski et al. 2018; Miyayama 2019). However, from the systematic review in this study, only one study involved 5 patients who underwent delayed intervention after ineffective LAG treatment, while the other 6 patients were cured with the single LAG treatment (Kortes et al. 2014). Hence, it is still questionable to estimate the real efficacy of the different intervention for postoperative LF while excluding the influence of iodized oil-based LAG from published articles. In prospect, it is worthwhile to explore which strategy is better: simultaneous iodized-oil LAG with sequential intervention, or iodized oil-based LAG with delayed intervention if the LF persists after LAG. In this case, it indicated that delayed intervention after ineffective iodized oil-based LAG might be a better choice with a higher cost-efficiency to treat the postoperative LF.

## Conclusions

Four major types of the interventions can be considered for postoperative LF at different locations, including direct leakage embolization/sclerotherapy (DLE/DLS), percutaneous afferent lymphatic vessel embolization (ALVE), percutaneous afferent lymphatic vessels disruption/sclerotherapy (ALVD/ALVS), and trans-afferent nodal embolization (TNE). Three potential lymphatic targets should be comprehensively assessed in prior iodized oil-based LAG, including confined leakage, definite afferent LVs, and definite closest afferent LNs. In addition, post-CT LAG is probably a useful complementary modality for dedicated treatment planning of interventions for postoperative LF meriting further exploration.

## Supplementary information

**Additional file 1.**

## Data Availability

All data were illustrated in this article and no additional data were available.

## Abbreviations

LF: Lymphatic fistula
LAG: Lymphangiography
ALVE: Afferent lymphatic vessel embolization
ALVD: Afferent lymphatic vessel disruption
ALVS: Afferent lymphatic vessel sclerotherapy
CLFD: Central lymphatic flow disorder
DLE: Direct leakage embolization
DLS: Direct leakage sclerotherapy
INL: Conventional intranodal lymphangiography
LN: Lymph node
LV: Lymphatic vessel
MIP: Maximum intensity projection reformatted imaging
MRL: Magnetic resonance-lymphangiography
NBCA: N-butyl cyanoacrylate
PLPS: Pulmonary lymphatic perfusion syndrome
Post-LAG CT: Post-lymphangiographic computed tomography
PVA: Polyvinyl alcohol
TDD: Percutaneous thoracic duct disruption
TDE: Percutaneous thoracic duct embolization
TL: Conventional trans-pedal lymphangiography
TNE: Trans-nodal embolization
3D-VR: 3-dimensional volume rendering
