# Supplementary material for: The roles of iodized oil-based lymphangiography and post-lymphangiographic computed tomography for specific lymphatic intervention planning in patients with postoperative lymphatic fistula: a literature review and case series
Source: CVIR Endovasc. 2020 Oct 21;3:79. doi: 10.1186/s42155-020-00146-x (PMC7578215; doi:10.1186/s42155-020-00146-x)
Supplement: Supplementary file 1 — Additional file 1. [file 42155_2020_146_MOESM1_ESM.docx]

**Electronic Supplementary Materials**

**Electronic Supplement 1**

**Search strategy**

((("Chylothorax"[Mesh] OR "Chylous Ascites"[Mesh] OR "Lymphocele"[Mesh] OR "Manual Lymphatic Drainage"[Mesh] OR (("Chyle"[Mesh] OR "Lymphatic Vessels"[Mesh]) AND ("Fistula"[Mesh] OR "Drainage"[Mesh] OR "Pericardial Effusion"[Mesh] OR "Pleural Effusion"[Mesh] OR "Ascites"[Mesh]))) OR ("Chylothorax"[All Fields] OR "Chylaskos"[All Fields] OR "Lymphocele"[All Fields] OR "Chylopericardium"[All Fields] OR (("Chylous"[All Fields] OR "Lymphatic"[All Fields] OR "Chyle"[All Fields] OR "Chylopericardial"[All Fields] OR "Chyluria"[All Fields]) AND ("Fistula"[All Fields] OR "Fistulas"[All Fields] OR "Leakage"[All Fields] OR "Ascites"[All Fields] OR "Effusion"[All Fields] OR "Effusions"[All Fields] OR "Drainage"[All Fields])))) AND ("Lymphography"[MeSH Terms] OR "Lymphography"[All Fields] OR "Lymphangiography"[All Fields])) AND ( "1999/05/13"[PDat] : "2019/05/13"[PDat] )

**Note:** search date – 13th May 2019.
